# Supplementary figures and images for: Bacteriophages isolated from mouse feces attenuates pneumonia mice caused by Pseudomonas aeruginosa
Source: PLoS One. 2024 Jul 16;19(7):e0307079. doi: 10.1371/journal.pone.0307079 (PMC11251617; doi:10.1371/journal.pone.0307079)

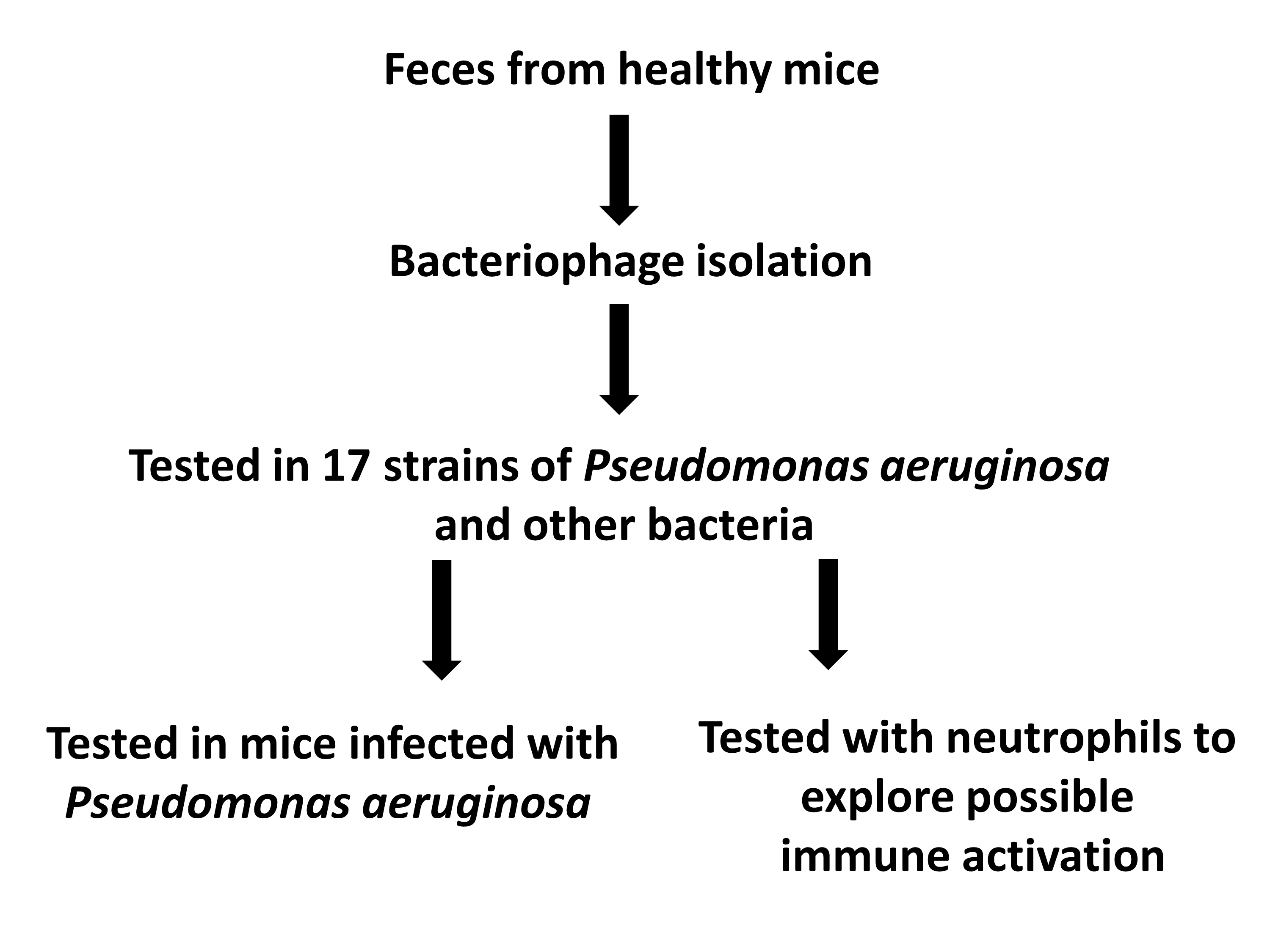

Supplement: S1 Fig — (TIF) [file pone.0307079.s001.tif]
